# Prévalence de l'hépatite B chez les personnes infectées par le VIH à Parakou au Bénin

**DOI:** 10.11604/pamj.2015.20.125.6061

**Published:** 2015-02-12

**Authors:** Comlan Albert Dovonou, Salimanou Ariyoh Amidou, Amadohoué Arsène Kpangon, Yacoubou Adam Traoré, Togbemabou Primous Martial Godjedo, Assongba Joseph Satondji, Ablo Prudence Wachinou, Fatioulaye Mahamadi Issa-Djibril, Léonard Fourn, Djimon Marcel Zannou, Prosper Gandaho

**Affiliations:** 1 Service de Médecine Interne, CHU de Parakou; 2 Département de Médecine et spécialités médicales de la Faculté de Médecine de l'Université de Parakou; 3 Centre d'Information de Prospectives et de Conseils sur les IST/VIH/Sida, Parakou; 4 Programme National de Lutte contre la Tuberculose, Cotonou, Bénin; 5 Département de Santé Publique, Faculté des Sciences de la Santé Cotonou; 6 Clinique Universitaire de Médecine Interne, CNHU-HKM de Cotonou; 7 Service de Psychiatrie, CHU de Parakou

**Keywords:** VIH, hépatite B, prévalence, Bénin, HIV, hepatitis B, prevalence, Benin

## Abstract

**Introduction:**

La co-infection avec l'hépatite B est l'un des défis majeurs de la prise en charge du VIH depuis l'amélioration de l'accès aux antirétroviraux en Afrique. La présente étude visait à estimer la prévalence de l'hépatite B chez les personnes séropositives au VIH à Parakou et décrire les facteurs associés.

**Méthodes:**

Il s'agit d'une étude transversale menée de Mai 2011 à Juin 2012 dans le service de Médecine du CHU de Parakou. Ont été inclus tous les adultes séropositifs au VIH vus en consultation ou hospitalisés. Les données ont été collectées par interviews et dépouillement de dossiers médicaux. L'antigène HBs a été recherché par un test rapide et l'ALAT a été dosé. L'analyse des données a été faite avec le logiciel EpiInfo 3.5.1. Les proportions ont été comparées grâce au test de Chi-deux ou au test de Fisher au seuil de significativité de 5%. Un modèle de régression logistique multivariable a permis d'expliquer la prévalence de l'hépatite B.

**Résultats:**

Sur les 744 sujets inclus on a dénombré 555 femmes. L’âge moyen était de 35,5 + 10,1 ans. La prévalence de l'hépatite B a été estimée à 16,9% (IC_95_: 14,3%-19,9%). Cette prévalence était plus élevée chez les sujets originaires du Borgou/Alibori et ceux au stade 4 de l'OMS.

**Conclusion:**

La prévalence de la co-infection VIH/VHB au CHU Parakou est élevée. Le dispositif national de prise en charge et de prévention de l'hépatite B chez les personnes séropositives au VIH doit être renforcé.

## Introduction

La pandémie du VIH est la plus grande qu'ait jamais connue le monde avec environ 34,2 millions de personnes infectées en fin 2011 [1]. L'infection par le VIH est aujourd'hui un problème de développement en Afrique subsaharienne. Elle y est associée à une prévalence élevée de l'infection au Virus de l'Hépatite B (VHB) [2], ceci étant en partie liée au partage des mêmes voies de transmission par ces deux affections. En 2009, on estimait à 2 milliards le nombre de personnes infectées par le VHB dans le monde [3]. L'Afrique est le premier continent touché par le VIH, et le deuxième pour les hépatites B juste derrière l'Asie [4]. L'hépatite B est souvent asymptomatique (60%) mais la forme aigüe de la maladie (40%) peut évoluer dans 0,1% à 1% des cas vers une hépatite fulminante, et dans 5 à 10% des cas vers la chronicité, qui favorise la cirrhose ou le cancer du foie d’évolution fatale. L'infection par le VIH modifie l'histoire naturelle du VHB et aggrave le pronostic de l'hépatite chronique B [5, 6]. Elle majore notamment le risque de passage à la chronicité en augmentant la réplication virale du VHB. Bien que chaque comorbidité soit un facteur aggravant potentiel de l’évolution de l'infection à VIH par l'affaiblissement de l'organisme qu'elle induit, l'hépatite B ne semble pas avoir une influence directe sur la progression de l'infection à VIH.

La découverte des antirétroviraux (ARV) a profondément modifié l’évolution de la pandémie du VIH en la transformant en une maladie chronique. L'accès aux ARV, quoique encore insuffisant, est une réalité en Afrique et permet d'améliorer tant la qualité que l'espérance de vie des personnes séropositives au VIH. De nouveaux défis s'imposent alors pour la préservation des bénéfices de ce traitement. Ces défis requièrent la prise en compte des comorbidités, en particulier la co-infection VIH/VHB. Mais l'insuffisance de données freine la riposte. A Cotonou au Bénin, une étude réalisée au CNHU en 2010 a rapporté une prévalence hospitalière du portage de l'Ag HBs de 11,21% chez les personnes vivant avec le VIH (PVVIH) sous ARV [6]. Par contre à Parakou, ville abritant la 2ème Faculté de Médecine du Bénin, aucune étude ne s'est encore intéressée à cette association morbide. Pour combler ce vide, la présente étude visait à estimer la séroprévalence du VHB chez les PVVIH suivis dans le Service de Médecine Interne du Centre Hospitalier Universitaire (CHU) de Parakou et identifier les facteurs associés à la co-infection.

## Méthodes

**Cadre d’étude**: l’étude a été réalisée dans le service de Médecine Interne du CHU de Parakou au Bénin. Ce service a enregistré du 1er Mars 2004 au 20 Juin 2011, 1447 patients infectés par le VIH dont 927 ont été traités par les ARV. Le nombre total de sujets effectivement en suivi était estimé à environ 1000 parmi lesquels 800 sont sous ARV. Les analyses de laboratoire ont été effectuées au Laboratoire du CIPEC-B/A qui est le laboratoire de référence du niveau départemental pour la biologie du VIH.

**Type et période**: il s'agissait d'une étude transversale. La collecte de données s’était déroulée sur la période du 16 mai 2011 au 30 Juin 2012.

**Population**: la population d’étude était composée de l'ensemble des personnes vivant avec le VIH (PvVIH) suivies dans le service de Médecine Interne du CHU de Parakou. Il a été inclus toutes les personnes séropositives au VIH, venues en consultation pour leur suivi ou hospitalisées durant la période d’étude, sous traitement ARV ou non, âgés de 15 ans au moins et ayant donné leur consentement éclairé.

**Collecte de données cliniques**: la collecte des données s'est déroulée en 3 étapes: entretien en face à face avec le patient pour collecter les données sociodémographiques et les antécédents; le dépouillement du dossier médical pour collecter les données cliniques de l’évolution de l'infection à VIH, les antécédents médicaux et les traitements donnés; enfin la prescription d'un bilan sanguin comportant la recherche de l'Ag HBs et le dosage des Alanine Amino-tranférase (ALAT). Le diagnostic de l'infection à VIH a été fait selon les normes en vigueur au Bénin, donc suivant la stratégie 2 de l'OMS. Elle a consisté en l'utilisation de deux tests: un test sensible (ELISA, test rapide Determine^®^) et un test discriminant (Génie 2^®^, Bioline^®^). Les sujets considérés comme séropositifs au VIH sont ceux ayant eu, un test sensible positif, puis confirmé par un test discriminant qui précise le type de virus (1 ou 2). L'Ag HBs a été recherché sur les prélèvements de sang veineux par le test rapide HB KIT^®^ de PLETHICO PHARMACEUTICALS LIMITED. Un contrôle de qualité a été également réalisé sur chaque 10ème prélèvement par le Laboratoire du Centre Départemental de Transfusion Sanguine par la méthode ELISA (Enzyme Linked Immuno Sorbent Assay) avec le réactif Monolisa HBs Ultra de BIO RAD^®^. Les Alanine Amino-Transférase (ALAT) ont été quantifiées par un semi-automate de biochimie de type Spectrophotomètre avec des réactifs ERBA^®^ Diagnostics Mannheim GmbH pour détecter une cytolyse. La norme pour cette méthode était: ALAT < 49 UI/l.

**Variables étudiées**: la variable dépendante en étude est la coinfection VIH + VHB, variable binaire codée: oui ou non. Elle avait la valeur oui lorsque la recherche de l'antigène de surface du virus de l'hépatite B (Ag HBs) est positive chez le sujet infecté par le VIH. Les expositions étaient: l’âge, le sexe, la situation matrimoniale, le département d'origine, le niveau d'instruction, l'homosexualité, la transfusion sanguine, l'Utilisation de Drogues Injectables (UDI), les pathologies hépatiques antérieures, les antécédents vaccinaux contre le VHB, la consommation habituelle d'alcool, le stade clinique OMS, les ALAT, le type de VIH, le taux de CD4 le plus récent, la valeur de charge virale la plus récente, le traitement ARV et les molécules utilisées.

### Traitement et analyse des données

Les données régulièrement vérifiées et corrigées ont été enregistrées et traitées à l'aide du logiciel EpiInfo^®^ 3.5.1. L'anonymat a été assuré par l'utilisation du numéro d'enregistrement du patient. La base de données a été contrôlée au fur et à mesure que la saisie progressait. L'analyse a d'abord porté sur la description du profil sociodémographique et clinique des sujets inclus. Les fréquences ont été estimées avec leurs intervalles de confiance et les moyennes avec leurs écarts-types. L'analyse du rapport entre la prévalence et les différentes caractéristiques de l’échantillon a été faite par la régression logistique. Un seuil de significativité de 5% a été utilisé. Cette étude a été conduite dans le respect des règles éthiques et de l'accord d'Helsinki.

## Résultats

Pendant la période d’étude, 918 sujets séropositifs au VIH ont été reçus dans le service de Médecine interne du CHU de Parakou. Sept cent quarante-quatre (744) fiches d'enquête complètes et exploitables ont été récupérées, soit un taux de participation de 81,0%. Les patients non inclus sont ceux qui ne s’étaient pas rendus au laboratoire avant la fin de la période d’étude et dont les résultats d'AgHBs n’étaient pas disponibles à temps.

### Caractéristiques sociodémographiques

Parmi les 744 sujets inclus, on dénombrait 555 femmes (74,6%) et 189 hommes (25,4%). La sex-ratio était de 0,34. Les sujets de moins de 45 ans représentaient 87,1% de l’échantillon. L’âge moyen était de 35,5+ 10,1ans et les extrêmes de 16 et 71 ans. La répartition des sujets suivant les différentes tranches d’âge est représentée à la figure N°1. Plus d'un tiers des patients sont originaires des départements de Borgou/Alibori (35,1%), suivi des départements limitrophes de l'Atacora/Donga (23,7%) et du Zou/Collines (23,0%). Selon leur situation matrimoniale les 744 sujets se répartissaient en 279 (37,5%) qui vivaient seuls, 176 (23,6%) avec un partenaire dont ils ignoraient le statut sérologique VIH, 156 (21,0%) dans un couple séropositif et 133 (17,9%) dans un couple sérodifférent. La majorité des sujets était donc en couple (62,5%). Parmi ces derniers (n = 465), la monogamie était le régime le plus fréquent, 76,9% (n = 371) contre 23,1% vivant sous un régime polygame. Aucun patient n'a déclaré l'utilisation de drogues injectables, ni chez les hommes des rapports sexuels avec un autre homme.

### Caractéristiques cliniques, biologiques et thérapeutiques

**Figure 1:**
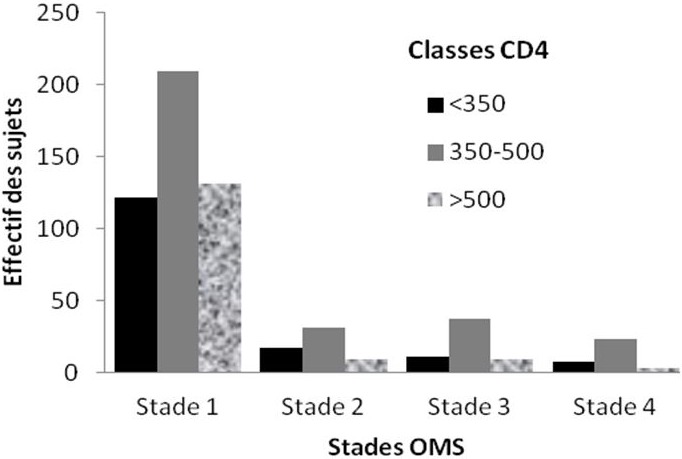
Distribution des 610 sujets ayant eu un dosage de CD4 en fonction du stade clinique OMS et du taux de CD4. Etude sur la prévalence de la coinfection VIH/VHB à Parakou en 2012

La quasi-totalité (97,6%) des sujets était infectée par le VIH1, 1,8% par le VIH2 et 0,6% par les 2 sérotypes à la fois. Les sujets étaient souvent classés au stade 1 (71%) de l'OMS. La numération des CD4 était disponible pour 622 patients. La valeur moyenne du nombre de CD4 était de 381,64 cellules/mm3. La répartition par classe du nombre de CD4 et par stade OMS est présentée à la Figure 1. Les résultats de la charge virale ont été obtenus pour 138 sujets dont 128 sous ARV. Elle a été indétectable 48 fois exclusivement chez les sujets sous ARV. Cinq cent huit (508) sujets étaient sous ARV. Le traitement le plus utilisé est la combinaison Zidovudine + Lamivudine + Névirapine (38,0%). La Lamivudine est associée à la quasi-totalité (504/508) des traitements institués tandis-que l'association Tenofovir + Lamivudine a été observée chez 7 patients dont 6 ayant déclaré des antécédents de co-infection VIH/VHB. L’étude des antécédents personnels médicaux des sujets avait révélé que la vaccination contre l'hépatite B était rare. Parmi les 6 patients vaccinés (au moins une dose), seuls 3 ont reçu les 3 doses requises. De même, 184 patients (25,2%) sont des consommateurs habituels d'alcool.

### Séroprévalence de la co-infection VIH + VHB

L'antigène de surface du virus de l'hépatite B (AgHBs) a été retrouvé chez 126 sujets sur les 744 inclus, soit une séroprévalence de la coinfection VIH + VHB estimée à 16,9% (IC95%: (14’ > IC95%: (14,3%-19,9%). Les ALAT ont été dosés chez tous les patients en même temps que la recherche de l'AgHBs. Ainsi, 90% des patients avaient le taux d'ALAT normal (N = ALAT < 49), 8,6% présentait une légère cytolyse (N < ALAT < 2N) et 1,4% une cytolyse modérée à sévère (ALAT > 2N)

### Les facteurs associés à la co-infection

En analyse univariable, le sexe, l’âge, la situation matrimoniale, le niveau d'instruction, le nombre de CD4, la charge virale détectable, la prise d'alcool, le taux d'ALAT et la prise d'ARV n’étaient pas statistiquement associés à la coinfection VIH/VHB (p > 0,05). L'appartenance géographique aux départements du Borgou/Alibori et le stade 4 OMS étaient associés à une prévalence plus élevée de l'hépatite B. Les résultats de la régression logistique univariable pour l'origine géographique et la classification OMS sont présentés dans le Tableau 1. L'analyse des résultats des modèles de régression logistique incluant ces deux dernières variables à la fois n'a mis en évidence aucune modification d'effet, ni confusion. Les résultats des modèles univariables ont donc été conservés.

**Tableau 1:** Facteurs associés à la co-infection VIH-VHB au CHD-Borgou en 2012, Résultats de la régression logistique univariable

	Ag HBs positif/négatif		
Facteurs évalués	RC estimé	IC _ 95% _ (RC)	P value
Sexe			
Féminin	1		
Masculin	1,39	[0,91-2,12]	0,1175
Age			
[15-24]	1		
[25-49]	1,03	[0,55-1,95]	0,9111
≥ 50	0,52	[0,20-1,34]	0,1777
*Classes OMS*			
Classe 1	1		
Classe 2	0,90	[0,45-1,78]	0,7682
Classe 3	0,87	[0,46-1,64]	0,6690
Classe 4	**2,52**	**[1,21-5,21]**	**0,0128**
Origine géographique			
Borgou/Alibori	1		
Atacora/Donga	0,60	[0,36-1,00]	0,0509
Zou/Collines	0,44	[0,25-0,76]	0,0037
Autres	0,54	[0,30-0,95]	0,0344

## Discussion

Cette étude a permis d'estimer la prévalence de la co-infection VIH-VHB au CHU de Parakou à Parakou et d'identifier quelques facteurs associés. L'inclusion exhaustive des sujets, la durée de l’étude (14 mois) et le taux élevé de participation ont permis de limiter les biais de sélection. Le type transversal de l’étude ne permettait cependant pas de tirer des conclusions sur les liens de causalité avec les facteurs associés identifiés. Il a été observé une prédominance féminine qui corrobore la féminisation de l'infection à VIH en Afrique subsaharienne [7], mais plus prononcée que les estimations de prévalences en population générale au Bénin en 2012 qui donnaient 1,4% pour les femmes et 1,0% pour les hommes [8]. Il y a donc une surreprésentation des femmes dans l’échantillon qui peut être due à un meilleur accès aux soins de santé ou une plus grande demande de soins chez les femmes. Elle est conforme aux tendances observées jusque-là dans le même service sur diverses pathologies [9, 10]. Les sujets étaient essentiellement des jeunes (87% de moins de 45 ans), avec une moyenne d’âge de 35,68 ans et des extrêmes de 16 et 71 ans. Cette distribution est comparable à l’épidémiologie du VIH au Bénin et partout en Afrique au sud du Sahara [11–14] où l’épidémie touche la force productrice. Les sujets originaires du Borgou/Alibori étaient les plus représentés, suivis des Zou/Collines et Atacora/Donga qui sont des départements limitrophes du Borgou. Ceci peut s'expliquer par la proximité et les liens séculaires entre les peuples de ces 3 départements dont les ressortissants sont les plus nombreux à Parakou. Le suivi de l'infection à VIH étant un suivi au long cours, la tendance générale est de se faire suivre dans son milieu de vie habituel tandis qu'une affection aigue peut motiver une consultation d'urgence, même loin de chez soi. Les comportements à risque persistent en général au sein de cette population de sujets séropositifs car bien que la majorité vivait en couple, certains sont dans des foyers polygames sans connaissance du statut du conjoint, ni partage du résultat positif. Il est donc nécessaire de renforcer l'intégration des programmes de prévention au sein des services de soins aux PvVIH, afin de réduire les risques de transmission du VIH (ou autres IST) à leurs partenaires d'une part et de réduire d'autre part les risques de surinfection VIH et de coinfection à d'autres IST pour PvVIH eux-mêmes.

Le portage de l'Ag HBs dans la population d’étude était de 16,9%. Le diagnostic de l'infection au VHB a été fait par la recherche de l'AgHBs. Ce moyen comporte un risque de minimisation de la prévalence en raison des hépatites B occultes dont la prévalence chez les PvVIH est estimée à 5% [15]. La détection de la charge virale à VHB ou la PCR auraient été les meilleures options pour prendre en compte les hépatites B occultes. Mais, quoique probablement sous-estimée, la prévalence observée reste élevée et confirme la répartition géographique de l'endémicité du VHB, selon laquelle la prévalence du VHB serait ‘ 8% dans la région Sub-saharienne [16]. Elle est comparable à la prévalence retrouvée par Sèhonou et al. [13] à Cotonou en 2007 (11,2% [IC95% 7,21 - 15,21]), à la plupart des estimations en Afrique subsaharienne [5, 12, 17–20]. Elle est plus élevée que celle retrouvée dans le nord de l'Afrique et sur les autres continents (4,2 à 10,4%) [21–26]. Ceci est bien en rapport avec les conclusions de Makuwa et Al. [27] au Congo qui ont démontré que l'absence de marqueurs du virus de l'hépatite B était corrélée à l'absence d'infection à VIH. Cependant, les tendances à Parakou méritent d’être confirmées par une étude en population générale. Comme à Cotonou en 2007 [13] aucune association significative n'a été mise en évidence entre le sexe, l’âge, la situation matrimoniale, le niveau d'instruction et la coinfection VIH/VHB. Elle touche donc autant les femmes que les hommes. Cependant, elle a été associée à l'appartenance aux départements du Borgou/Alibori. Il serait alors intéressant d'approfondir cette observation pour la confirmer ou non, et le cas échéant explorer les variations socioculturelles locales qui pourraient expliquer ce résultat en comparaison avec les autres départements. Il pourrait s'agir des pratiques culturelles telles que les scarifications, le lévirat, le sororat, l'excision, la circoncision qui n'ont pas été explorées dans la présente étude. En effet, Adewole et al. [28] avait observé au Nigeria comme facteurs de risque exposant à la co-infection, le multi-partenariat sexuel, les scarifications et les transfusions sanguines répétées. De même, pour Sèhonou et al. [13] les facteurs de risque exposant de façon significative à la co-infection étaient la transfusion sanguine et l'acupuncture, tandis-que Koike et al. [29] retrouvaient comme facteurs de risque exposant, l'usage de drogues injectables (UDI), la transfusion sanguine et l'homosexualité masculine. Ces derniers facteurs n'ont pu être explorés ici car aucun cas d'Homme ayant des rapports Sexuels avec des Hommes (HSH), d'UDI n'a été déclaré. Ces deux sous-populations sont en effet peu nombreuses au Bénin: en 2012, une enquête exploratoire a dénombré 1382 HSH répartis dans 13 grandes villes du Bénin et 35 UDI essentiellement à Cotonou [30]. Bien que les actions en direction de ces cibles se renforcent au Bénin, leur absence dans l’échantillon est aussi le reflet des pesanteurs sociales source de stigmatisation à l'origine de difficultés d'accès aux soins. Le stade 4 OMS de l'infection à VIH a été associé à une prévalence plus élevée de la co-infection. Elle pourrait aussi être liée à la forte probabilité pour un sujet à un stade clinique avancé de l'infection à VIH de passer au portage chronique plutôt que d’éliminer le VHB après la primo-infection. Ce constat également observé par Adewole et al [28] au Nigeria pourrait aussi être le reflet d'une plus longue durée d'exposition de ces sujets aux facteurs de transmission communs aux deux virus, accumulant ainsi le risque d’être infecté par l'un et l'autre. Ce sont autant d'hypothèses dont l'exploration permettrait de mieux comprendre la coinfection VIH/VHB. La cytolyse hépatique (ALAT > 2N) n’était pas statistiquement associée à la coinfection mais elle était plus marquée chez les coinfectés (4,9%) que chez les autres sujets (1%).

Aucun des patients vaccinés n’était infecté, mais l'effectif était trop faible pour en tirer des conclusions statistiques. De même la prévalence de l'infection au VIH2 était trop faible pour en explorer le rapport avec la coinfection. La Lamivudine, également active sur le VHB a été utilisée dans la quasi-totalité des traitements antirétroviraux. Mais la récurrence de la résistance du VHB à la Lamivudine a motivé l'adoption de l'association Tenofovir + Lamivudine chez les sujets coinfectés VIH/VHB. Cette association a été retrouvée chez 7 patients dont 6 coinfectés, donc 100% des sujets antérieurement dépistés pour le VHB dans cet échantillon ont été mis sous Tenofovir + Lamivudine. Cette proportion descend à 4,7% si l'on tient compte du total des coinfectés. Le dépistage est donc le maillon faible de la mise en æuvre de la politique de prise en charge de la coinfection. Le dépistage systématique de l'hépatite B garantirait donc une meilleure prise en charge de cette comorbidité pour en prévenir les complications.

## Conclusion

La prévalence de l'Ag HBs chez les PVVIH suivies dans le service de Médecine Interne du CHU de Parakou est de 16,9%. Elle était plus élevée chez les sujets au stade 4 de l'OMS et ceux originaires des départements Borgou/Alibori.
